# Congenital adrenal hyperplasia. Role of dentist in early diagnosis

**DOI:** 10.1515/med-2022-0524

**Published:** 2022-10-27

**Authors:** Michele Di Cosola, Francesca Spirito, Khrystyna Zhurakivska, Riccardo Nocini, Roberto Lovero, Salvatore Sembronio, Luigi Santacroce, Edoardo Brauner, Giovanni Storto, Lorenzo Lo Muzio, Angela Pia Cazzolla

**Affiliations:** Department of Clinical and Experimental Medicine, University of Foggia, Foggia, Italy; Department of Medicine, Surgery and Dentistry “Scuola Medica Salernitana,” University of Salerno, Via Allende, 84081, Baronissi (SA), Italy; ENT Department, University of Verona, 37100, Verona, Italy; Clinical Pathology Unit, AOU Policlinico Consorziale di Bari - Ospedale Giovanni XXIII, 70124, Bari, Italy; Department of Maxillofacial Surgery, University of Udine, Academic Hospital “Santa Maria della Misericordia,”, 33100, Udine, Italy; Department of Dental and Maxillary Facial Sciences, “Sapienza” University of Rome, 00185, Rome, Italy; Nuclear Medicine Unit, IRCCS Regional Cancer Hospital CROB, Referral Cancer Center of Basilicata, 85028, Potenza, Italy; Department of Interdisciplinary Medicine, Microbiology and Virology Laboratory, University Hospital of Bari, Università degli Studi di Bari, 70124, Bari, Italy

**Keywords:** CAH, adrenogenital syndrome, oral manifestations, oral findings, gingiva, teeth

## Abstract

Congenital adrenal hyperplasia (CAH) is a genetic disorder characterized by an impairment of steroid synthesis due to an altered production of 21-hydroxylase enzyme. Corticoid hormones are involved in the development and functioning of many organs. The aim of the present study was to review the international literature to collect data regarding oral manifestations of CAH. A review of the literature describing oral features of patients affected by CAH was performed using electronic databases (PubMed and Scopus). The data about number of patients, form of CAH, and oral findings were extracted and analyzed. Seven studies were included in the final analysis. The principal findings reported regarded an advanced dental development observed in patients with CAH. One paper reported amelogenesis imperfecta and periodontal issues. The dentist could be the first specialist involved in the CAH syndrome diagnosis, identifying the characteristic features described above, especially for the classical simple virilizing and non-classical form.

## 1 Introduction

Congenital adrenal hyperplasia (CAH) is an autosomal recessive disorder that, in the vast majority of cases, develops due to a mutation of sequence of the CYP21A2 gene. Such mutation leads to 21-hydroxylase (21-OH) deficiency that results in poor cortisol production and accumulation of precursor steroid hormones in the steroidogenic pathway [1,2]. Clinically, the manifestations range from mild to severe forms, distinguished as non-classical and classical CAH. The classical CAH occurs in about 1:15,000 live births and can assume two phenotypes: simple virilizing (SV) and salt wasting. The latter represents the most severe manifestation, in which a major shortage of 21-OH activity leads to an inadequate production of glucocorticoids and mineralocorticoids. The lack of aldosterone, if left untreated, can be incompatible with life, since it is fundamental for mineral salts homeostasis [3]. If the 21-OH activity is adequate to produce a sufficient amount of aldosterone, the CAH can manifest as SV (25% of all classical forms). In females, high levels of adrenal-derived androgens affect the development of the external genitalia, leading to their virilization, while males with SV form usually present an early virilization. A precocious pseudo puberty and hypocortisolism are also characteristics of both sexes [4].

Non-classical forms of CAH (NCAH) are characterized by around 20–50% of normal enzyme function and clinical manifestations are variable [5]. Patients may be asymptomatic at birth and primarily manifest symptoms at any age with signs of androgen excess. Frequently, the disease manifests with precocious pseudo puberty or polycystic ovary syndrome, hirsutism, and anovulation [5,6]. The therapies aim to address two issues: first, to replace deficient hormones, and second, to reduce excessive androgen levels.

In addition to the abovementioned manifestations, the organs and systems reported to be mainly influenced by such hormonal imbalance involve hypothalamus, cardiovascular system, bones, reproductive apparatus, musculoskeletal system, and skin [7].

Oral tissues are strongly influenced by steroid hormones. In particular, sex hormone receptors are widely present in the gingival tissue whose homeostasis is influenced by the blood fluctuation of these hormones [8]. Sex hormones group has been demonstrated to regulate cellular proliferation and differentiation in several tissues, among which keratinocytes and fibroblasts of the gingiva [9,10,11]. Estrogens increases the inflammatory component of gingiva, while progesterone plays a role in regulating the vascular permeability of the gingival and periodontal tissues, through the increase of prostaglandins production and polymorphonuclear leukocytes chemotaxis [12].

Several studies investigated the alteration of oral mucosa, gingiva, and periodontal tissue during the fundamental phases of hormonal development (i.e., puberty, menstrual cycle, pregnancy, and menopause in females), showing an important correlation between hormonal status and oral health [13].

In specific periods of woman’s hormonal development, when the estradiol and progesterone levels are the highest, an increase in inflammation and gingival bleeding has been noted, not correlated to the value of the plaque index which often culminates during gestation in the formation of a painless exophytic mass with a sessile or pedunculated base, defined as a “gravidic tumor” [9].

The aim of the present work is to review the existing literature to summarize the current knowledge about oral manifestations in patients with CAH in order to determine if any peculiar signs are associated and if the dentist could play a role in early diagnosis

## 2 Methods

This review was performed in order to answer the following question: “What are oral manifestations of CAH, if any?”

Electronic databases PubMed and Scopus were screened in order to search studies suitable for inclusion in this review. The following search strategy was used: [“congenital adrenal hyperplasia” AND (tooth OR teeth OR dental OR gingival OR oral manifestation OR oral mucosa)], [“adrenogenital” AND (tooth OR teeth OR dental OR oral manifestation OR oral mucosa)]. In addition, bibliographies of included studies were manually checked in order to identify other articles to be considered in this study. Only studies in English language and fulfilling the following criteria were eligible for inclusion:

– Studies reporting patients’ data with confirmed diagnosis of CAH.
– All study designs were considered (case reports, original papers, reviews, and conference proceedings).
– No restrictions about the year of publication were applied.

The articles, resulting from search strategy, were screened by title and abstract by two authors. If data reported in abstract were not sufficient for decision making, the full text reading was performed. So selected papers were full text examined and, if accordant with inclusion criteria, were included in the review. Any disagreement between authors was solved in a discussion.

The following data were extracted from the included studies:

– Authors’ names, year of publication, type of study design, number of patients, diagnosis, form of CAH, oral findings, and therapy.

## 3 Results

After application of search strategy, 181 records were identified. After removing duplicates, 63 records were screened by title and abstract evaluation and, subsequently, 10 were selected for full text examination. Two studies were excluded because they were written in non-English language [14,15], and for one study [16], only abstract was available. At the end, seven studies were included in the review [17,18,19,20,21,22,23]. Extracted data are summarized in Table 1.

**Table 1:** Data extracted from included studies

Authors	Year	Study design	Number of patients	Diagnosis	Classical or non-classical form	Oral findings	Therapy
Bergstrand and Filipsson [17]	1967	Congress proceedings	22 (13 M and 9 F)	Adrenogenital syndrome	Classical	Dental development is advanced in children with adrenogenital syndrome, but it is less influenced by androgens than skeletal development	Cortisone
Wagner et al. [18]	1963	Original study	7 (2 M and 5 F)	Congenital adrenocortical hyperplasia and adrenogenic virilism	Classical	Advanced dental development in children with congenital adrenocortical hyperplasia and androgen virilism	Cortisone
Garn et al. [19]	1965	Original study	6 (2 M and 4 F)	Virilizing adrenal hyperplasia	Not indicated	Advanced dental development	Not indicated
Roberts et al. [20]	1985	Original study	9 (8 M and 1 F)	Congenital adrenal hyperplasia	Not indicated	No significant abnormal dental development was detected	Not indicated
Singer et al. [21]	2001	Case report	1 F	Non-classic adrenal hyperplasia		Early shedding of the primary teeth	Hydrocortisone
Ajlan [22]	2015	Case report	1 F	Congenital adrenal hyperplasia	Classical	Amelogenesis imperfecta (AI), localized aggressive periodontitis	Prednisone and fludrocortisone
Angelopoulou et al. [23]	2015	Case report	1 M	Congenital adrenal hyperplasia	Classical	Premature exfoliation of primary teeth, accelerated eruption of permanent dentition, tooth mobility, bone destruction	Hydrocortisone and potassium levothyroxine

The data available in the literature resulted to be very scarce. The most recent studies [21,22,23] were case reports, presenting single cases of CAH patients and their oral features. The other included papers were original studies, but date back to the 60 s [18,19] and 80 s [20]. One of the papers was a congress proceeding extract [17]. However, even in the original studies, the sample of included patients was small.

Terms used in the studies to identify patients with CAH were: “Adrenogenital syndrome” [17], “Congenital adrenocortical hyperplasia” [18,20,21,22,23], “Adrenogenic virilism” [18], and “Virilizing adrenal hyperplasia” [19]. Regarding oral findings in patients with CAH, five studies reported an advanced dental development, [17,18,19,21,23] even if they noted that it was accelerated less than the acceleration of skeletal maturation observed in young patients. A study by Roberts et al. [20] concluded that no significant difference was observed between the affected patients and control group in terms of dental development. Meanwhile, one study [22] reported a case with other oral alterations: amelogenesis imperfecta and periodontitis in a patient with CAH.

## 4 Discussion

CAH is a genetic disorder resulting from an alteration of hormone production in the adrenal gland. It can result from the failure of any of the enzymes involved in the cascade of steroid hormone synthesis but in the vast majority of cases, it is caused by a deficiency of 21-OH [24]. The responsible gene is CYP21A2, for which over 100 mutations leading to various phenotypes have been reported [25,26,27]. Among these, the variant characterized by mutation 841G > T has been characterized and can be found in the CYP21A2 database created by the Human Cytochrome P450 (CYP) Allele Nomenclature Committee (https://www.pharmvar.org/htdocs/archive/cyp21.htm) [28]. The clinical phenotype reported for such mutation is the NCAH, with an *in vitro* activity of 17-Hydroxyprogesterone/Progesterone of 50%/20%. The residual 21-OH enzyme activity, in such patients, leads to androgen excess, but also to some cortisol and aldosterone deficiency [6,29,30]. NCAH forms are not usually characterized by virilization of genitalia and diagnosis is typically made in adolescence or adulthood [5,31]. In most of the reported cases, the first diagnosis was made after the onset of premature pubarche and hirsutism [31], confirmed by specific hormonal dosage and genetic analysis [5]. The prevalence of NCAH varies widely for different ethnicities, being reported to affect about 0.1% of Caucasian population and reach 3.7% in Ashkenazi Jews [32].

Regarding the treatment of NCAH, it has been suggested that a stimulated cortisol level of less than 500 nmol/L may justify daily glucocorticoid supplementation [33].

Steroid hormones represent a fine regulator of various functions of our organism [34]. And their imbalance, as well as a substitution therapy, can lead to various undesirable consequences, more or less serious. In non-classical forms, fortunately, the damage of the various organs and functions is limited, compared to the classical form [35,36,37].

Oral cavity, with its hard and soft tissues, is often the site of manifestation of several systemic and genetic diseases [38,39,40,41,42]. The role of sex steroid hormones on periodontal tissues has been widely documented [9,11,13,43]. Androgen receptors have been identified in human oral mucosa by Ojanotko-Harri et al. [44] in 1992, indicating their location in epithelial cells, fibroblasts, and endothelial cells. Estrogen is the main sex steroid hormone responsible for alterations in blood vessels of target tissues in females [45]. The variation in its levels also affects the vascularization of the periodontal tissues, exposing women to para-physiological conditions of the periodontium in periods such as puberty, ovulation, pregnancy, and many other causes [13]. Progesterone has been demonstrated to lead to the accumulation of inflammatory cells in blood vessels, increase vascular permeability and increase vascular proliferation. The presence of steroid receptors on immune system components has been identified in several studies [9,46].

All these data suggest that a disease such as CAH can also lead to oral manifestations.

The present review aimed to identify all existing information about oral manifestations in patients with CAH. However, the data in the literature were very scarce and there is lack of original studies with an adequate sample to be able to draw any conclusion. It appears that in children with CAH, there is a tendency for their dentition to develop early. Although in most of the studies included in this review, there is no clear identification of the classical and non-classical forms, it can be assumed that the more severe oral manifestations are characteristic of the classical forms [17,18,22,23].

Considering the demonstrated influence of steroid hormones on inflammation state of the periodontal tissues and therefore the possible increased risk of developing periodontitis in patients with CAH, further studies are certainly needed to investigate the role of early inflammatory biomarkers in CAH patients.

The dentist could be the first specialist involved in the CAH syndrome diagnosis, identifying the characteristic features described above, especially for the classical SV and non-classical form.
